# Potential Obesogen Identified: Fungicide Triflumizole Is Associated with Increased Adipogenesis in Mice

**DOI:** 10.1289/ehp.120-a474a

**Published:** 2012-12-03

**Authors:** Valerie J. Brown

**Affiliations:** Valerie J. Brown, based in Oregon, has written for *EHP* since 1996. In 2009 she won a Society of Environmental Journalists’ Outstanding Explanatory Reporting award for her writing on epigenetics.

Obesogens are chemicals that increase either the number of fat cells in an organism or the amount of fat stored in those cells. Obesogens may also act indirectly on obesity by modulating appetite, satiety, or metabolism. Now researchers have identified a common agricultural chemical that appears to qualify as an obesogen because it nudges gene expression and stem cell differentiation toward becoming a fat cell [*EHP* 120(12):1720–1726; Li et al.].

More than two-thirds of the U.S. population—twice the global average—is either overweight or obese. This is typically attributed to overeating and inactivity. But evidence that pets, laboratory animals, primates, and feral cats living in industrialized human societies also are showing a rise in obesity suggests that environmental obesogens may be playing a role.

In the current study, investigators exposed human and mouse mesenchymal stem cells (MSCs) and preadipocytes to triflumizole (TFZ), a fungicide widely used on food and ornamental crops. MSCs can differentiate into bone, cartilage, or fat cells; preadipocytes are precursor fat cells that mature into adipocytes in response to environmental cues. The investigators found that expression of obesity-related genes increased in treated cells from both species, and that lipid accumulation and expression of obesity-related genes increased in treated cells from both species.

**Figure:**
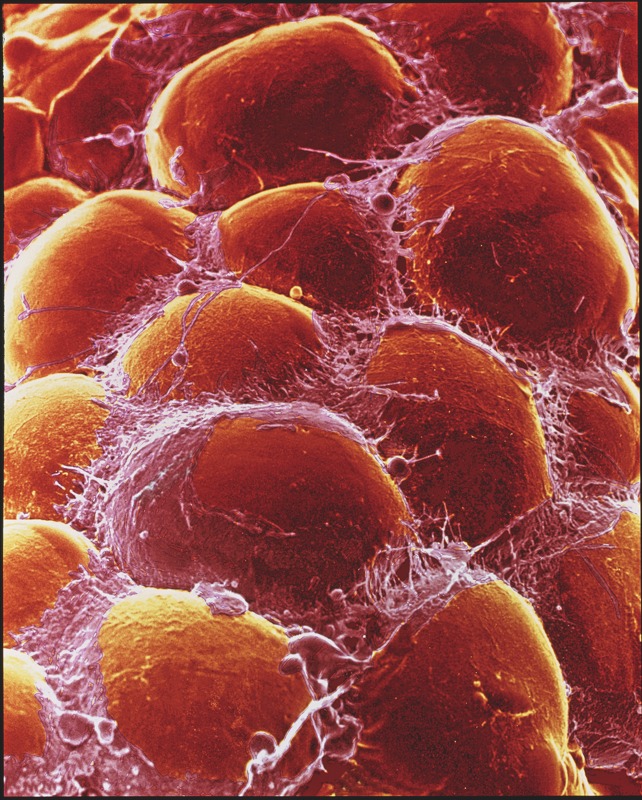
As adipocytes fill with lipids from the diet and increase in number, fat mass grows. © 2012 Quest/Photo Researchers, Inc.

The team then exposed three groups of pregnant mice to three different doses of TFZ and examined fat tissues and gene expression profiles of the offspring. Although exposed and unexposed offspring did not differ significantly in body weight, mice exposed to the lowest dose of TFZ prenatally showed increased mass of the “fat depot” (areas where fat is stored) compared with unexposed offspring. In contrast, fat depot mass did not differ from controls in animals treated with higher doses of TFZ. The lowest dose was 400 times lower than the established no-observed-adverse-effect level for TFZ exposure in rodents.

The authors observed that induced cultured MSCs from the exposed offspring appeared to be more likely to become fat cells than bone cells. TFZ has been identified as an activator of peroxisome proliferator-activated receptor gamma (PPARγ), which is known to regulate fat cell differentiation and gene expression. Treatment of exposed MSCs with a PPARγ blocker stopped their differentiation into fat cells, confirming that TFZ operates by this pathway.

Although the exposed offspring were of normal weight when they were sacrificed at 8 weeks, the authors suggest that changes in body weight might have increased with time, had the mice aged naturally. The increased fat mass in prenatally exposed mice was observed at exposure levels likely to be encountered by the general human population, according to the investigators (no actual data exist for human exposure to TFZ). They suggest that future studies should include biomonitoring of TFZ levels in humans and investigation of transgenerational effects and epigenetic mechanisms related to the chemical’s potential influence on obesity.

